# Enhancement of Vaccination Attitude and Flu Vaccination Coverage among Pregnant Women Attending Birthing Preparation Course

**DOI:** 10.3390/vaccines9020183

**Published:** 2021-02-21

**Authors:** Stefania Bruno, Brigida Carducci, Gianluigi Quaranta, Viria Beccia, Andrea Di Pilla, Daniele Ignazio La Milia, Marcello Di Pumpo, Elettra Carini, Lucia Masini, Enrica Tamburrini, Antonietta Spadea, Gianfranco Damiani, Antonio Lanzone, Patrizia Laurenti

**Affiliations:** 1 Women, Children and Public Health Sciences Department, Fondazione Policlinico Universitario A. Gemelli IRCCS, 00168 Rome, Italy; stefania.bruno@unicatt.it (S.B.); brigida.carducci@policlinicogemelli.it (B.C.); gianluigi.quaranta@unicatt.it (G.Q.); danieleignazio.lamilia@policlinicogemelli.it (D.I.L.M.); lucia.masini@policlinicogemelli.it (L.M.); enrica.tamburrini@policlinicogemelli.it (E.T.); gianfranco.damiani@unicatt.it (G.D.); Antonio.Lanzone@unicatt.it (A.L.); patrizia.laurenti@unicatt.it (P.L.); 2 Dipartimento di Scienze della Vita e Sanità Pubblica, Università Cattolica del Sacro Cuore, 00168 Rome, Italy; viria.beccia01@icatt.it (V.B.); marcello.dipumpo01@icatt.it (M.D.P.); elettra.carini01@icatt.it (E.C.); 3 Local Health Authority, ASL ROMA 1, District 14, 00135 Rome, Italy; antonietta.spadea@aslroma1.it

**Keywords:** awareness, flu vaccinations, on-site vaccinations, birthing preparation course, pregnant women

## Abstract

Most vaccinations are recommended within the 15th month of life, in order to reduce risks and to protect children from the initial stages of their lives. A vaccination training session was carried out during the birthing preparation course, aimed at increasing the attitude toward vaccination in maternal-child age. A questionnaire on vaccination awareness was administered before and after the training session and on-site flu vaccination was offered to women and their companions. The percentage of participants who consider the preparatory course a useful tool to obtain information about vaccines increases significantly from 30.34% at pre-intervention to 64.56% at post-intervention (*p* < 0.001). There is a significant increase in the mean number of vaccinations that the participants want their children to get. The number of participants believing that there is no relationship between vaccination and autism rose from 41.05 to 72.97% (*p* < 0.001). In total, 48 out of 119 (40.34%) pregnant women participating in the course and 39 companions were vaccinated for influenza. Vaccination knowledge and attitude significantly increased after a training session dedicated to vaccination as a part of the pregnant pre-birth course, whose aim can be therefore extended to the management of the health of the child, well beyond the period of pregnancy, according to the life-course approach to health.

## 1. Introduction

Vaccination is the most effective and powerful tool against preventable infectious diseases. In order to protect children from the initial stages of their life, most vaccinations are recommended within the 15th month of life. In Italy, the law provides 10 compulsory vaccinations to the 0–16 age group and underlines the importance of vaccinations for women of child-bearing age and pregnant women [1]. Particularly, according to the World Health Organization (WHO), the first at-risk category for influenza are pregnant women, because of the multiple effects on both mother and fetus [2]. Mandatory attention to pregnant women is highlighted by the Circular of 21 November 2018 (Italian Ministry of Health) that recommends flu vaccination to all women in the second or third trimester at the beginning of the flu season [1]. The Advisory Committee on Immunization Practices (ACIP) recommends that each pregnant woman, regardless of the trimester of pregnancy, should receive a flu vaccine [3]. Despite this, the vaccination coverage rate among pregnant women is usually lower than what is recommended, even with divergences among rates of different countries (from 0.3 to 56.1% in European countries, and 50.3% in the U.S.A. [4]. Several reasons explain low vaccination coverages, not only for flu. The primary reason is vaccine hesitancy, which is a multifactorial complex problem, whose main determinants is the level of knowledge about vaccines safety, efficacy, and side effects, compared to disease clinics and complications [5]. Pregnant women are therefore the ideal target for educational strategies, because their choices will affect their health and that of their children [6,7]. A vaccination training course was carried out during the birthing preparation course at a teaching hospital in Rome, primarily aimed at increasing awareness and attitudes to vaccination in pregnant women. The secondary aim of the study was to evaluate the effectiveness of the on-site influenza vaccination offer for pregnant women (and their partners), who became aware of herd immunity and its importance during the course. 

## 2. Materials and Methods

This study is compliant with the Local Ethical Committee Standards of the Fondazione Policlinico Universitario Agostino Gemelli IRCCS (FPG). It was approved and registered (Prot. N° 38264/19 ID: 2782) and was carried out in accordance with the Helsinki Declaration and EU Regulation 2016/679 (GDPR). For this kind of study, the Ethical Committee foresaw the need for participant consent. 

A before–after monocentric cross-sectional study was carried out in the teaching hospital FPG; the timeframe under observation runs from October 2019 to January 2020, during the influenza epidemic season. The methodology used is in accordance with the most recent Guidelines for Observational Studies, Strengthening the Reporting of Observational Studies in Epidemiology (STROBE) [8]. 

The population included in the study was represented by those attending the birthing accompaniment course that took place at the FPG during the months of October, November, December 2019, and January 2020. The course was organized by the Obstetrics and Obstetric Pathology Unit of the Department of Women’s and Children’s Health and Public Health, on a monthly basis and with weekly meetings and targeted at women from the 4th month of pregnancy and their companions. There were no exclusion criteria. No sample size was required: all women who participated in the course during the reporting period and consented to the study were therefore included; for this reason, the sample is a convenience one. 

A training session lasting from thirty to forty minutes in the birthing preparation course was carried out to raise awareness about vaccination during both pregnancy and puerperium and in early childhood. The birthing accompaniment course was made up of six monthly meetings of about two hours each; the proposed contents covered obstetrics, neonatology, and dental hygiene, in addition to the session dedicated to public health. The topics addressed in the vaccination session concerned: vaccines definition and functioning, vaccine constituents and categories, public health successes, adverse reactions, myths, vaccinations schedule during pregnancy, and vaccination access through the italian National Health System (NHS). Moreover, the vaccination calendar was presented with its epidemiological and biological rationale, highlighting the mandatory vaccines in Italy.

For the evaluation of the primary endpoint, a voluntary anonymous questionnaire about vaccination awareness was administered before and after the training session and changes in knowledge and awareness of vaccination were evaluated as a result of the training intervention. The pre and post questionnaires were identical and was previously used and validated in a multicentric Italian study, the NAVIDAD study [9,10]. The full questionnaire is included in the annexes. In our study, the questionnaire was used with different aims, as it was not administered as part of a Pre-Childbirth Course during flu vaccination campaign; moreover, no compliance to the flu vaccination was evaluated. The questionnaire was administered in a different setting within the Department of Obstetrics of an Italian University General Hospital. In addition, the purpose of our study was different, in fact, compliance through flu vaccination on site was evaluated after the training course.

The data obtained from the questionnaires were entered into a database created using Excel 2016 software. The categorical variables were properly converted into discrete numerical variables and the answers have been coded in numbers; the answers not given correspond to no numbers, as well as the answers given on 2 different options. In order to protect the anonymity of the answers and in view of the different number of participants who answered the questionnaire before and after the intervention, data were not paired.

For the evaluation of the implementation of the knowledge, the database data corresponding to the post questionnaires were compared to the pre set. The categorical variables were described in terms of absolute and relative frequencies (percentages). The continuous variables were studied for normality using the Shapiro–Wilk test. The distributions of the continuous variables were described in terms of mean and standard deviation. 

The evaluation of knowledge implementation and attitudes variation in the pre and post training questionnaires was carried out with the Student T test, for continuous variables, and with the Chi-square test, for qualitative variables. Statistical significance was set at *p* = 0.05. The analysis was carried out using the software “Stata IC 14.2 for Mac” (Stata Corp, Lakeway, TX, USA; Single-user Stata perpetual license: 301406262870 Daniele La Milia). 

For the assessment of the secondary endpoint, the possibility for the women participating in the course and their companions to access on site influenza vaccinations at the end of the course session or on other planned dates was foreseen: six dates were scheduled in November (also dedicated to October participants), December and January. Vaccination was carried out free of charge and after collecting the medical history and informed consent. The effectiveness of the vaccination strategy was evaluated using the vaccination compliance data of pregnant women and their companions. Vaccination compliance of pregnant women was obtained by the proportion of the vaccinated pregnant women who participated in the course out of the total number of pregnant participants. The vaccination compliance of accompanying persons was obtained by the ratio of the vaccinated accompanying persons to the women participating in the course. Moreover, the total number of pregnant women vaccinated for influenza through the dedicated vaccination clinic during the reporting period was compared with the total number of pregnant women vaccinated at the gynecological obstetrician outpatient clinic (GOC) only, on the same period of the previous year. 

Pregnant women vaccinated on the days of the vaccination sessions who did not attend the training meeting during the course were not taken into account in the calculation of vaccination compliance. On the contrary, pregnant women who participated in the course and received vaccination at the GOC were included, the vaccination was provided and administered after the training session. Data analyzed for the compliance study were collected anonymously using Excel 2016 software and presented in aggregate form. The forms used for the collection of informed consent and medical history are included in the annexes. 

## 3. Results

Out of 119 pregnant women who attended the birthing preparation course from October 2019 to January 2020, equal to the sum of the 28 women participating in the birthing accompaniment course in October 2019, 44 participants in November 2019, 23 participants in December 2019 and 24 participants in January 2020. 

The questionnaires completed before the vaccine training intervention were 104, those completed after the intervention were 79. General characteristics of the women participating in the course and included in the study are shown in Table 1. 

Regarding the intention to vaccinate the child, 103 pre-intervention women and 77 post-intervention women, equal to the totality of the pregnant women who answered the questionnaire (100%), have positively answered (1 missing in the pre- and 2 missing in the post-intervention questionnaires). Concerning diseases to vaccinate the child, out of 12 infectious diseases as response options, women in the pre-intervention questionnaires scored an average of 9.68 options, while in the post-intervention questionnaires 10.57 options, equal, respectively, to 80.67 and 88.08% of the response options, showing a significant increase (*p* = 0.021).

Analyzing the preferences expressed for vaccinations for the individual infectious diseases proposed, results were obtained with statistically significant differences between the pre- and post-intervention questionnaires only in relation to *tetanus* and HPV (*Human Papilloma Virus*), as shown in Table 2.

As regards to how to acquire information on vaccines before the training intervention, 42 women out of 104 (40.38%) reported they received information from health personnel, 62 denied it, and 55 women searched for the information themselves (52.88%).

In relation to the usefulness of information channels for obtaining information on vaccinations, statistically significant differences were obtained for the available information channels with regard to Local Health Authority or Ministry of Health information brochures and birthing preparatory course. In the pre-intervention questionnaire, 30 women out of 82 (36.59%) considered the information brochures to be of no use at all, and 27 women out of 89 (30.34%) considered the childbirth preparation course to be very useful. In the post-intervention questionnaires, 16 out of 75 women (21.33%) considered the information brochures to be of no use at all, and 51 out of 79 women (64.56%) considered the preparatory course to be very useful, showing a significant reduction and increase, respectively, (*p* = 0.052 and *p* < 0.001). 

With regard to opinions on NHS, health workers, and vaccinations, statistically significant differences were obtained, only for the following two on “NHS operators are prepared and updated on vaccinations” and “NHS operators give information only of the benefits and not of the risks of vaccines”. Out of 101 pre-intervention questionnaires (3 missing data), 35 women (34.65%) strongly agreed with the statement that NHS workers are prepared and updated on vaccinations, while this was stated by 46 women in the 79 post questionnaires (58.23%), confirming a significant increase (*p* = 0.007). With reference to the statement that NHS operators do not give information on the risks of vaccinations, in the pre-intervention questionnaires (4 missing data), 31 women out of 100 (31.00%) reported they did not agree at all, equally 38 women out of 79 (48.00%) in the post-intervention questionnaires, confirming a significant increase (*p* = 0.029).

In relation to the knowledge of the epidemiology of childhood infectious diseases, out of 12 diseases considered, no statistically significant differences were found between pre- and post-intervention responses. On the contrary, in relation to the knowledge of the severity of childhood infectious diseases, out of 12 infectious diseases considered, statistically significant differences were obtained for *Haemophilus influenzae b, poliomyelitis,* and *diphtheria.*

*H. influenzae* infection was considered quite severe by 31 out of 87 women (35.63%) in pre-intervention questionnaires and 40 out of 74 women in post-intervention questionnaires (54.05%, *p* = 0.051). *Poliomyelitis* infection was considered very severe by 64 out of 93 women (68.82%) in the pre-intervention questionnaires and 69 out of 78 women in the post-intervention questionnaires (88.46%, *p* = 0.005). *Diphtheria* infection was considered very severe by 36 out of 89 women (40.45%) in the pre-intervention questionnaires and 47 out of 76 women in the post-intervention questionnaires (61.84%, *p* = 0.020).

Regarding the opinion of compulsory vaccination for school enrollment, 97 out of 101 (96.04%) women agreed in the pre-intervention questionnaires and 78 out of 79 women in the post-intervention questionnaires (98.73%, *p* = 0.387).

About the impact of some claims on vaccines and their safety profile on the choice to vaccinate their children and the opinion on general information on vaccines and their safety, results are shown in Table 3. 

During the study, 48 out of 119 (40.34%) pregnant women participating in the course were vaccinated for influenza (46 on-site vaccinations and 2 at the GOC), 39 companions were also vaccinated on-site (average age 39.44 ± 7.19). Through the on-site vaccinations 5 pregnant women, not made aware through the training intervention were also vaccinated. The total of pregnant women vaccinated for influenza through the on-site vaccination was 51 (average age 35.02 ± 4.55, average gestation week 31.93 ± 3.68). Moreover, 26 women were vaccinated at the GOC, therefore the total number of pregnant women vaccinated for the 2019–2020 season was 77, whereas in the previous year the total number was 63, all at the GOC.

## 4. Discussion

### 4.1. Principal Findings

The study has shown the increase in knowledge and attitudes of the participants following a training intervention on vaccinations. Women became aware that vaccines are basically safe, with an excellent benefit–risk ratio, which, according to several studies [11,12,13,14,15,16,17,18,19] is not perceived, and becomes one of the determinants of vaccine hesitancy. The information provided has increased the knowledge of the severity of diseases that can be prevented by vaccines, in a statistically significant way, with particular reference to *Haemophilus influenzae b, poliomyelitis, diphtheria*, confirming that the impact of vaccinations has reduced the incidence and prevalence of these diseases, reducing the perceived severity, too. Vaccine coverage in Italy is above the 95% optimal threshold for *polio* and *diphtheria*, slightly lower for *H. influenzae b* [20]. Understanding the severity of these diseases can improve vaccination coverage in a conscious way, changing perspectives, from a duty to a right [15].

Another important aspect of the study was the possibility to address some unfounded myths related to vaccination, such as too many vaccinations are administrated in children too young. After the training intervention, there was an increase of more than a third (31.87%, *p =* 0.005) in the number of women who understand that vaccinations are not carried out on children too young was obtained [21]. The importance of the vaccination schedule was well understood by the women who participated in the course, in fact, there was an increase of 18.18% (*p =* 0.011) in the group of those who recognized the vaccination schedule as a method designed for children’s protection. In addition, an increase of 12.52% (*p =* 0.038) of women who recognized the vaccination as a tool to protect other children who cannot be vaccinated was obtained. Thus, the importance of herd immunity was well understood, and the vaccination was recognized not only as an individual protection tool, but as responsibility towards the community as well [22]. The data from the accompanying people highlights how awareness of vaccination is effective as a means of protecting the community in its fundamental unit, the family. By disseminating the information received, women played the role of catalysts, aware of the benefits of herd immunity, the importance of which was highlighted during the training meeting.

Among the main unfounded beliefs against vaccinations, the issue that vaccines cause autism still unfortunately exists: data shows an important increase (77.76%) of women who recognize the absence of this association (pre 41.05%, post 72.97%, *p <* 0.001), highlighting the necessity of focusing efforts on the dissemination of correct, scientifically proven information aimed at eradicating myths and erroneous beliefs [21,23]. The study results were encouraging, demonstrating the importance of the information provided. The intervention was able to increase knowledge and remove hesitancy about vaccines, and this finding is similar to that of another Italian study [24]. On this aspect, the data reported confidence on the reliability of the information sources and the trust of participants in the NHS operators are also of great interest [25]. The training intervention gave the opportunity of an in-depth discussion on vaccines and the opinion that the health workers offering vaccinations are very well prepared increased by 68.05% (*p =* 0.007).

Influenza vaccination among pregnant women significantly reduces hospitalization rates and maternal respiratory insufficiency [26], and stillbirths [27], prematurity and underweight for gestational age births [28,29], infections of high airways, and otitis media for children [1]. Moreover, the on-site campaign proved to be a valid tool to vaccine, as highlighted in previous studies [30,31,32]; the coverage achieved in the study is excellent if compared to the Italian vaccination coverage in the general population for the 2018–2019 influenza season (15.8%), although it is not possible to compare it with the collected Italian data for pregnant women because they are not recorded [20]. D’Alessandro highlights a self-reported coverage for influenza among pregnant women of 1.4% [17].

### 4.2. Strengths and Limitations

Nevertheless, among the study limitations, filling out the questionnaires anonymously did not allow comparison for data matched. Moreover, no data were collected for flu vaccination administered to pregnant women at other supply points. Furthermore, not pairing the data and the reduction in the number of answers in the questionnaire administered after the course may have generated a bias in the possibility that more motivated and vaccination-oriented people answered the questionnaire after the course. However, it is possible to state that for the variables collected, the composition of the group of participants who answered the first questionnaire is not statistically different from the composition of the group of participants who answered the second questionnaire (e.g., with regard to the participants’ level of education, 77.88% of those who answered the first questionnaire were university graduates, compared with 78.48% of those who answered the second, *p* = 0.93). Confounding factors, such as vaccination policies or public opinion, which may have affected knowledge and attitudes to vaccination at the same time of the intervention, were not controlled in statistical analysis.

Regarding the sample size, it represents a limitation of the study: the Authors had no possibility to select the number of women and increase it to obtain a larger sample. All the women who attended the course at the Obstetrician Department were recruited. For this reason, the sample size depends on the number of women who attended the course. Anyway, as the course and the flu vaccination campaign have been repeated during the flu season 2020–2021, a subsequent study will allow to increase and expand the sample size. At this moment, the study represents an initial experience and the studied sample is one of convenience.

Moreover, the sample of women who answered the questionnaire after the intervention is different from the sample of women who answered the questionnaire before.

This mismatch is due to the fact that some women who fulfilled the questionnaire before the intervention did not fill in the questionnaire after the same intervention because they were not present during the same intervention.

For all these reasons the two sample sizes are not the same (the number of the women is different), but the sample is basically represented by the same women.

On the other hand, one of the strengths of the study is the multidisciplinary (gynecologists, public health, and infectious diseases physicians) and multi-professional collaboration with patients on a crucial issue of public health and maternal and child health. 

This study can be a starting point and a reference point for the evaluation and enhancement of the availability of influenza vaccination of pregnant women, through the active and dedicated offer, also on site, which can be accessed through various clinical-assistance paths. The WHO recalls that the most effective strategies in improving attitudes and increasing knowledge and winning vaccination wavering include the introduction of educational initiatives, included in dedicated protocols and pathways, which also facilitate access to vaccinations themselves [5,33]. 

## 5. Conclusions

The recent SARS-CoV-2 pandemic also highlighted the importance of influenza vaccination, especially in the winter months, when the simultaneous circulation of two respiratory viruses showed uncertainty about epidemiological, diagnostic, and therapeutic differentiation. Particularly in a pandemic period, when the allocation of resources may be conditioned by temporary needs, the WHO stresses, nevertheless, the importance of maintaining normal vaccination activities as much as possible, in order to prevent this from leading to an accumulation of susceptible people and an increased risk of vaccine-preventable disease outbreaks, adding new problems to pre-existing ones, and old problems to new ones [34]. 

The key message is that immunization is vital to prevent serious diseases and protect health and well-being at all ages and that awareness of the importance of vaccines for health protection can be increased through dedicated, evidence-based programs; finally, the dedicated on-site vaccination sessions appear as a useful public health tool in order to improve the vaccination compliance and reduce the supply-side immunization barriers.

## Figures and Tables

**Table 1:** General characteristics of the women taking part in the course included in the study.

Variables	Before Intervention; N (%)	Post Intervention; N (%)	*p*-Value
Italian citizenship	98/103 * (95.15)	72/79 (91.14)	0.28
Married	103/104 (99.04)	79/79 (100.00)	0.85
Degree	81/104 (77.88)	62/79 (78.48)	0.93
Employee	51/104 (49.04)	40/79 (50.63)	0.83
Freelance	24/104 (23.08)	17/79 (21.52)	0.80
Healthcare worker	14/104 (13.46)	12/79 (15.19)	0.74
Job Seeking	4/104 (3.85)	4/79 (5.06)	0.69
Housewife	3/104 (2.88)	3/79 (3.80)	0.73
Student	2/104 (1.92)	2/79 (2.53)	0.78
Craftswoman	1/104 (0.96)	1/79 (1.27)	0.84
Manager	1/104 (0.96)	0/79 (0.00)	0.85
Other Occupation	4/104 (3.85)	0/79 (0.00)	0.29
First Pregnancy	100/104 (96.15)	76/79 (96.20)	0.99
Third Trimester	90/104 (86.54)	71/79 (89.87)	0.49
Age (mean ± SD **)	34.49 ± 4.91	34.77 ± 5.12	？

* Denominator corresponds to the number of women who answered this question. ** SD: Standard Deviation.

**Table 2:** Preferences expressed for vaccinations to the individual infectious diseases proposed.

Infectious Diseases	Before Intervention; N (%)	Post Intervention; N (%)	*p*-Value
Tetanus	84/104 (80.77)	72/79 (91.14)	0.050
HPV	53/104 (50.96)	52/79 (65.82)	0.044
Hepatitis B	91/104 (87.50)	73/79 (92.41)	0.281
Poliomyelitis	79/104 (75.96)	67/79 (84.81)	0.140
Haemophilus influenzaeb	61/104 (58.65)	56/79 (70.89)	0.082
Diphtheria	81/104 (77.88)	69/79 (87.34)	0.099
Pertussis	99/104 (95.19)	73/79 (92.41)	0.432
Measles	98/104 (94.23)	76/79 (96.20)	0.541
Rubella	89/104 (85.58)	74/79 (93.67)	0.082
Parotitis	84/104 (80.77)	65/79 (82.28)	0.795
Meningitis	91/104 (87.50)	68/79 (86.08)	0.777
Varicella	87/104 (83.65)	69/79 (87.34)	0.486

**Table 3:** Opinion on general information on vaccines and their safety and impact of some claims on vaccines and their safety profile on the choice to vaccinate their children (the denominator corresponds to the number of women who have answered this question).

Claims	Before Intervention; N (%)	Post Intervention; N (%)	*p*-Value
Vaccines have mild side effects	76/98 (77.55)	76/78 (97.40)	0.002
Adverse reactions of vaccines do not only depend on the antigenic component	34/96 (35.42)	58/78 (74.36)	<0.001
Vaccines are sufficiently tested before being placed on the market	77/97 (79.38)	74/78 (94.87)	0.008
Vaccinations are not carried out on children too young	64/97 (65.98)	68/78 (87.01)	0.005
Immune system does not struggle to manage multiple vaccinations simultaneously	47/98 (47.96)	58/78 (74.36)	0.002
Vaccination calendar is designed to protect children	76/96 (79.17)	73/78 (93.56)	0.011
Diseases cannot be prevented only by following healthy lifestyles, without vaccination	79/96 (82.29)	75/79 (94.87)	0.039
Vaccinating your own child protects other children as well	84/97 (86.60)	76/78 (97.44)	0.038
There’s no relationship between vaccines and autism	39/95 (41.05)	54/74 (72.97)	<0.001
It is false that the disease for which one is vaccinated is often less dangerous than the vaccine itself	69/95 (72.63)	65/75 (86.67)	0.055
Vaccines are carried out on children who are too young: no impact on the vaccination choice	34/93 (36.56)	40/74 (54.04)	0.001
Immune system struggles to manage multiple vaccinations simultaneously: no impact on the vaccination choice	29/90 (32.22)	36/73 (49.32)	0.010
Vaccinating your own child protects other children as well: great impact on the vaccination choice	54/91 (59.34)	59/77 (76.62)	0.016
The side effects of vaccines are kept hidden: no impact on the vaccination choice	29/88 (32.95)	33/73 (45.21)	0.022
The disease for which one is vaccinated is often less dangerous than the vaccine itself: no impact on the vaccination choice	36/87 (41.38)	40/70 (57.14)	0.049

## Data Availability

The data presented in this study are available on request from the corresponding author.
